# Anti-inflammatory and anti-diabetic properties of indanone derivative isolated from *Fernandoa adenophylla* in vitro and in silico studies

**DOI:** 10.1038/s41598-024-59703-2

**Published:** 2024-04-26

**Authors:** Abdur Rauf, Umer Rashid, Zafar Ali Shah, Anees Ahmed Khalil, Muhammad Shah, Tabussam Tufail, Gauhar Rehman, Abdur Rahman, Saima Naz, Abdulrahman Alsahammari, Metab Alharbi, Abdulmajeed AL-Shahrani, Dorota Formanowicz

**Affiliations:** 1 Department of Chemistry, University of Swabi, Swabi, Anbar, 23430 Khyber Pakhtunkhwa (KP) Pakistan; 2 Department of Chemistry, COMSATS University Islamabad, Abbottabad Campus, Islamabad, 22060 Pakistan; 3 Department of Agricultural Chemistry and Biochemistry, The University of Agriculture, Peshawar, Pakistan; 4 University Institute of Diet and Nutritional Sciences, Faculty of Allied Health Sciences, The University of Lahore, Punjab, Pakistan; 5 Department of Zoology, Abdul Wali Khan University, Mardan, Khyber Pakhtunkhwa (KP) Pakistan; 6 Institute of Biotechnology and Microbiology, Bacha Khan University, Charsadda, Khyber Pakhtunkhwa (KP) Pakistan; 7 Department of Pharmacology and Toxicology, College of Pharmacy, King Saud University, Post Box 2455, 11451 Riyadh, Saudi Arabia; 8 Laboratory Department, Almadah General Hospital, Ministry of Health, Khamis Mushait, Saudi Arabia; 9 Chair and Department of Medical Chemistry and Laboratory Medicine, Poznan University of Medical Sciences, 60-806 Poznan, Poland; 10 Department of Stem Cells and Regenerative Medicine, Institute of Natural Fibres and Medicinal Plants, National Research Institute, Kolejowa 2, 62-064 Plewiska, Poland

**Keywords:** *Fernandoa adenophylla*, Anti-inflammatory, Anti-diabetic, In silico study, Biochemistry, Cell biology, Chemical biology, Computational biology and bioinformatics, Drug discovery

## Abstract

*Fernandoa adenophylla*, due to the presence of phytochemicals, has various beneficial properties and is used in folk medicine to treat many conditions. This study aimed to isolate indanone derivative from *F. adenophylla* root heartwood and assess in-vitro anti-inflammatory and anti-diabetic characteristics at varying concentrations. Heat-induced hemolysis and glucose uptake by yeast cells assays were conducted to evaluate these properties. Besides, docking analyses were performed on four molecular targets. These studies were combined with molecular dynamics simulations to elucidate the time-evolving inhibitory effect of selected inhibitors within the active pockets of the target proteins (COX-1 and COX-2). Indanone derivative (10–100 µM) inhibited the lysis of human red blood cells from 9.12 ± 0.75 to 72.82 ± 4.36% and, at 5–100 µM concentrations, it significantly increased the yeast cells’ glucose uptake (5.16 ± 1.28% to 76.59 ± 1.62%). Concluding, the isolated indanone might act as an anti-diabetic agent by interacting with critical amino acid residues of 5′ adenosine monophosphate-activated protein kinase (AMPK), and it showed a binding affinity with anti-inflammatory targets COX-1, COX-2, and TNF-α. Besides, the obtained results may help to consider the indanone derivative isolated from *F. adenophylla* as a promising candidate for drug delivery, subject to outcomes of further in vivo and clinical studies.

## Introduction

Herbal plants have gained recognition as a valuable repository of bioactive phytochemicals, extracted, refined, and employed in creating various contemporary medications^1^. Compared to synthetic drugs, natural products derived from plant metrics have gained popularity among consumers. These products have captured the interest of researchers due to their favorable attributes, such as safety, reduced toxicity, and cost-effectiveness, compared to numerous synthetically developed drugs^2^. Identifying medicinal compounds derived from crude plants has prompted the pharmaceutical industry to allocate resources toward ethnomedicinal research, which is vital in developing their formulations^3^. Traditional usage of medicinal plants provides preliminary information regarding health-promoting benefits associated with them and, therefore, possesses a crucial role in drug development^4^. Studies comprising the phytochemical composition, in-vitro and in-vivo biological activities, and pharmacological screening have positively aided the drug development industry^5^. Various phytochemicals (pistagremic acid, diospyrin, punicalagin, 8-hydroxyisodiospyrin, etc.) have been identified and isolated from several plant species. They have effectively treated different ailments (diabetes, inflammation, infections, fever, cancer, hyperlipidemia, etc.)^6–8^. Due to their health benefits and reduced toxicity, the pharmaceutical industry is allocating a substantial budget towards researching and developing drugs that incorporate naturally derived biologically active plant components^9^.

Scientists have extensively examined numerous medicinal plants with well-documented therapeutic properties. However, there remains a multitude of unexplored and underutilized plant species that hold potential for medicinal applications. One such plant is *Fernandoa adenophylla*; hence, the current study was designed to investigate bioactivities scientifically. *Fernandoa adenophylla*, also recognized as *Haplophragma adenophyllum*, is a member of the Bignoniaceae family and belongs to the genus Fernandoa. This flowering plant is most abundantly present in various regions of Southeast Asia, like Bangladesh, India, Thailand, Andaman Islands, and Vietnam. Locally, this plant is known by different names, such as Lotum-poh, Karen wood, Mostan-phul, Dhopa-phali, and Ziron^6^. *Fernandoa adenophylla* is a deciduous tree having a height ranging from 5–13 m and is also found in North America and Africa. Genus *Fernandoa* is of ecological significance and comprises 860 species^10^. In the folkloric medicine system, *F. adenophylla* has been used by people in Thailand, Bangladesh, and India to treat skin-related problems, snake bites, constipation, and hemorrhoids^6^. In the subcontinent countries, various components of this plant have traditionally been recommended for treating infections, sepsis, premature and nocturnal ejaculations, amenorrhea, and skin issues^11^. In the ancient Thai medicine system, the leaves of this plant were utilized for treating skin-related ailments, while fruits and flowers were consumed after cooking and as fresh vegetables, respectively. In the case of viper bites, this plant's roots were ingested as a drink^11^. *Fernandoa adenophylla* (FA, *Heterophragma adenophyllum*) is a plant cultivated throughout Africa and Southeast Asia. It contains potent phytochemicals such as novel naphthoquinones, their derivatives (peshwaraquinone, dilapachone, adenophyllone, indadone, and lapachol), and triterpenoids (ursolic acid (UA), β-sitosterol (BS), α-amyrin, and oleanolic acid (OA)) that have been assessed and reported to show potential pharmacological activities^12^. The crude extract obtained from the plant has been investigated for certain pharmacological activities such as antibacterial, antifungal, anti-diabetic, anti-inflammatory, anti-tubercular (TB), antihypertensive, and leishmanicidal activity^13^.

Several crude extracts derived from *F. adenophylla* have been documented to exhibit antibacterial, antifungal, and leishmanicidal properties and antihypertensive and anti-TB activities^14^. It contains various phyto-molecules, i.e., lapachol, peshwaraquinone, dehydro-lapachone, β-amyrin, dilapachone, dehydro-iso-lapachone, β-sitosterol, ursolic acid, α-lapachone, oleanolic acid, adenophylone, and tecomoquinone-I^15^. Among all these above-stated naphthoquinones and triterpenoids, lapachol has been reported to be the most abundant phytochemical in most species found in the genus Bignoniaceae. Several bioactivities, such as anti-inflammatory, antimalarial, anti-carcinogenic, anti-abscess, anti-ulcer, anti-endemic, termiticical, and viricidal activities, have all been reported for lapachol and its derivatives^16,17^. Similarly, phytochemicals like α-lapachol, lapachol, indanone derivative, and peshawaraquinone have also shown inhibitory properties against Phosphodiesterase 1 and inflammation^18^. Considering the immense potential in the realm of medicinal plants, fueled by the diverse properties of *Fernandoa adenophylla*, there remains a scarcity of research in this domain, thereby presenting an opportunity for scientists to conduct further analyses. The existing literature indicates limited studies conducted on phytochemicals derived from Fernandoa adenophylla. Consequently, this knowledge gap served as the motivation behind the present study's design. Depending upon the therapeutic potential of this underutilized plant, the current study was designed to explore the anti-diabetic and anti-inflammatory potential of the indanone derivative isolated from *F. adenophylla.*

## Results

### Isolation and characterization of Indanone derivative

Indanone derivative was isolated as a white solid having melting points 161–163 °C. The EI-MS indicated a molecular ion peak at m/z 290.133 [M^+^] with molecular formula C_16_H_18_O_5_. Our research group previously identified the Indanone derivative's chemical structure using ^1^H-NMR and C^13^-NMR spectroscopy^11^.

### Effect of indanone derivative on heat-induced hemolysis

**Figure 1:**
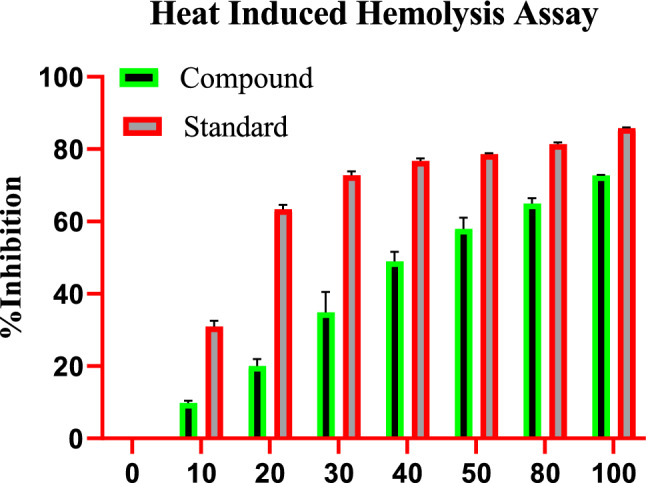
Effect of indanone derivative on heat-induced hemolysis assay.

High temperature results in the lysis of red blood cells (RBC), while the impact of indanone derivative on RBC stabilization was assessed in this study. Indanone derivative momentously aided in inhibiting heat-induced lysis of the RBC membrane in a dose-dependent manner; diclofenac sodium was used as the standard drug. Various concentrations of indanone derivative were used at 10, 20, 30, 40, 50, 80, and 100 µM, which showed 9.12 ± 0.75%, 17.97 ± 1.05%, 28.44 ± 1.84%, 45.91 ± 0.42%, 54.65 ± 3.51%, 63.21 ± 1.72%, and 72.82 ± 4.36% of inhibition respectively. The compound expressed 54.69 as the IC50 value. For the standard group, the diclofenac sodium was used at 10, 20, 30, 40, 50, 80, and 100 µg, which showed 32.66 ± 1.93%, 64.23 ± 1.34%, 73.80 ± 2.88%, 77.38 ± 3.12%, 78.57 ± 0.81%, 80.95 ± 4.64%, and 85.71 ± 2.89% of inhibition respectively. The minimum rate inhibition of the indanone derivative was at 10 µM, which was 9.12 ± 0.75%. While the maximum rate of inhibition was shown at 100 µM, which was 72.82 ± 4.36%, as shown in Fig. 1.

### Effect of different concentrations of indanone derivative on glucose uptake by yeast cells in glucose solution

**Figure 2:**
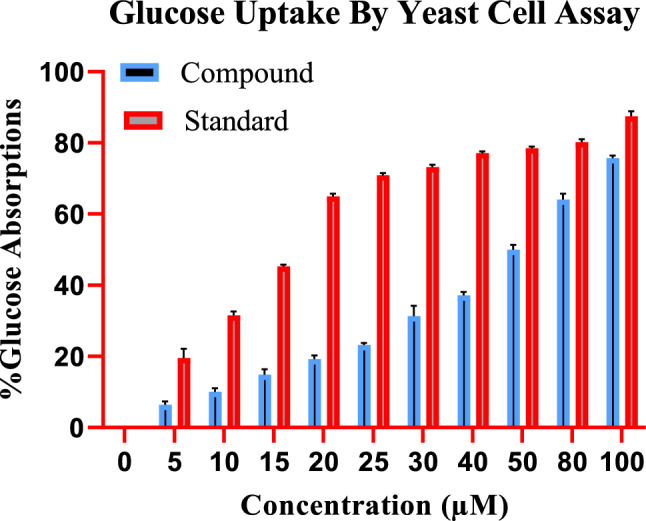
Effect of indanone derivative on glucose uptake by yeast cells in glucose solution (Glucose Yeast Uptake Assay/5 mM).

To study the effect of indanone derivative on the glucose uptake by yeast cells in 5 mmol glucose solution, various concentrations of indanone derivative (from 5 to 100 µM) were incubated. The compound has increased the uptake from 5.16 ± 1.28% to 76.59 ± 1.617% in the yeast cell at 5 µM and 100 µM concentrations, as shown in Fig. 2, respectively, compound reveals a dose-dependent increase in uptake.

The standard drug (Metformin) also has a pronounced effect on glucose uptake by the yeast cells, with an increase from 21.45 ± 0.88% to 88.65 ± 0.42% in glucose uptake at 5 µg and 100 µg concentrations of metformin, respectively. Figure 2 shows that the activity of the indanone derivative has followed the standard.

### Alpha-amylase inhibition assay

**Figure 3:**
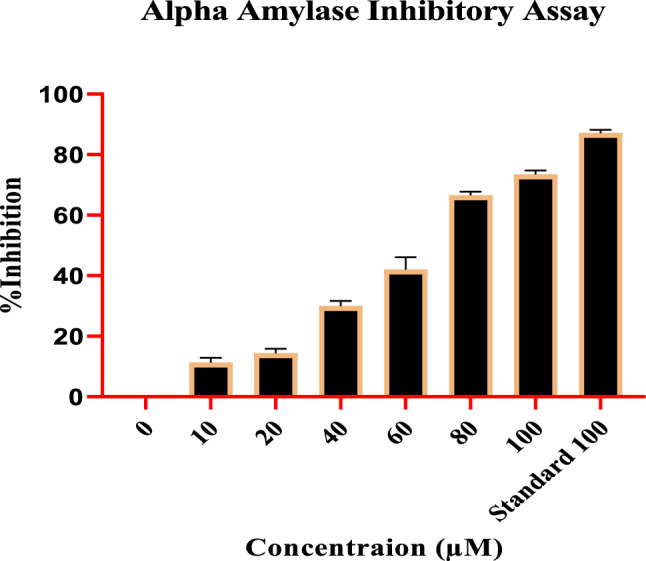
Compound effect on α-Amylase inhibitory potential at various concentrations.

Remarkable results were obtained assessing the Compound impact on the α-amylase inhibitory activity at various concentrations. The inhibitory potential of the compound was determined with different concentrations, i.e., 10, 20, 40, 60,80, and 100 (µM), for which inhibition percentage was recorded as 9.80 ± 0.54%, 13.07 ± 1.25%, 28.90 ± 1.87%, 46.12 ± 2.00%, 65.88 ± 1.64%, and 72.85 ± 1.19% respectively, with IC50 value of 46.37 µM. The standard drug, acarbose at 100 µg, expressed the highest inhibition at 87.08 ± 1.328%. With the increase in concentrations, a regular rise in the α-amylase inhibition was observed, indicating dose-dependent inhibition. Figure 3 shows that the activity of the compound has followed the standard.

### Molecular docking

We docked the indanone derivative into the binding site of four molecular targets responsible for diabetic and inflammatory activities. The target enzymes were downloaded from the protein data bank (PDB) and docked using Molecular Operating Environment (MOE 2016.08) Software. 5′ Adenosine monophosphate-activated protein kinase (AMPK), a key target to design drugs against obesity, metabolic syndrome, and type 2 diabetes, was downloaded with PDB ID 3AQV. While anti-inflammatory targets, two cyclooxygenase enzymes (COX-1 and COX-2), and tumor necrosis factor-alpha (TNF-α) were downloaded, PDB accession codes 1EQG, 1CX2, and 2AZ5, respectively. Prepared enzymes and native ligands were redocked into the binding sites of their respective enzymes. The purpose is to validate the docking protocol. Only validated docking procedures (RMSD < 2.0 Å) were adopted for further docking runs. The computed RMSD values for experimental and redocked native ligands in the binding sites of downloaded enzymes have been presented in Table 1.

**Table 1:** Computed RMSD values in Å between experimental and redocked ligands.

S. No.	PDB ID	Native ligand name/ID	RMSD (Å)
1	1EQG	Ibuprofen	0.96
2	1CX2	SC-558	0.93
3	2AZ5	307	1.16
4	3AQV	TAK	1.08

**Figure 4:**
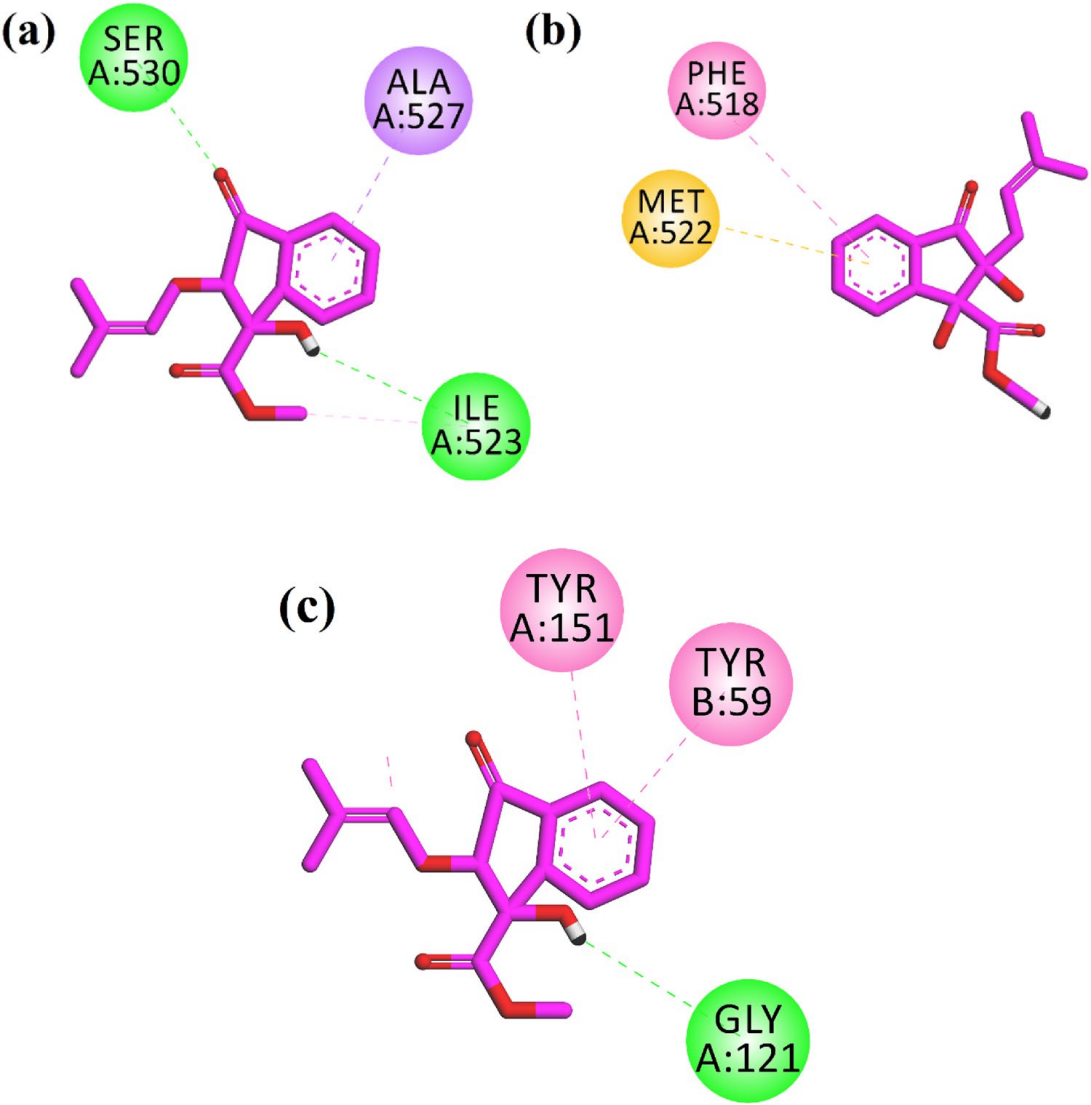
2-D interaction plots of isolated indanone into the binding site of (**a**) COX-1 (1EQG), (**b**) COX-2 (PDB ID = 1CX2) and (**c**) TNF-α (PDB ID = 2AZ5).

Two-dimensional (2-D) interaction plots of indanone into the binding sites of anti-inflammatory targets COX-1, COX-2, and TNF-α are shown in Fig. 4. The studied compound interacts with amino acid residues in the binding site COX-1 via 2 hydrogen bond interactions. Ile523 and Ser530 form hydrogen bond interactions with a hydroxyl group and carbonyl oxygen, respectively (Fig. 4a). Ile523 is a specific residue in the binding site of COX-1. The computed binding energy values of the native ligand and indanone derivative in the binding site of COX-1 are − 6.28 and − 5.48 kcal/mol, respectively. While in the binding site of COX-2, indanone derivative oriented deep into the binding site of COX-2 specific binding site residues Phe518 via π–π stacking interactions and π–sulfur interactions with Met522 (Fig. 4b). The computed binding energy values of the native ligand and indanone derivative in the binding site of COX-2 are − 9.65 and − 7.51 kcal/mol, respectively. A 2-D interaction plot of the compound in the binding site of TNF-α is shown in Fig. 4c. The hydroxyl group forms hydrogen bond interactions with Gly121, while Tyr59 and Tyr151 form π–π stacking interactions with the phenyl ring of the indanone (Fig. 4c). The computed binding energy values of native ligand and indanone derivative in the binding site of TNF-α − 7.08 and − 6.71 kcal/mol, respectively.

**Figure 5:**
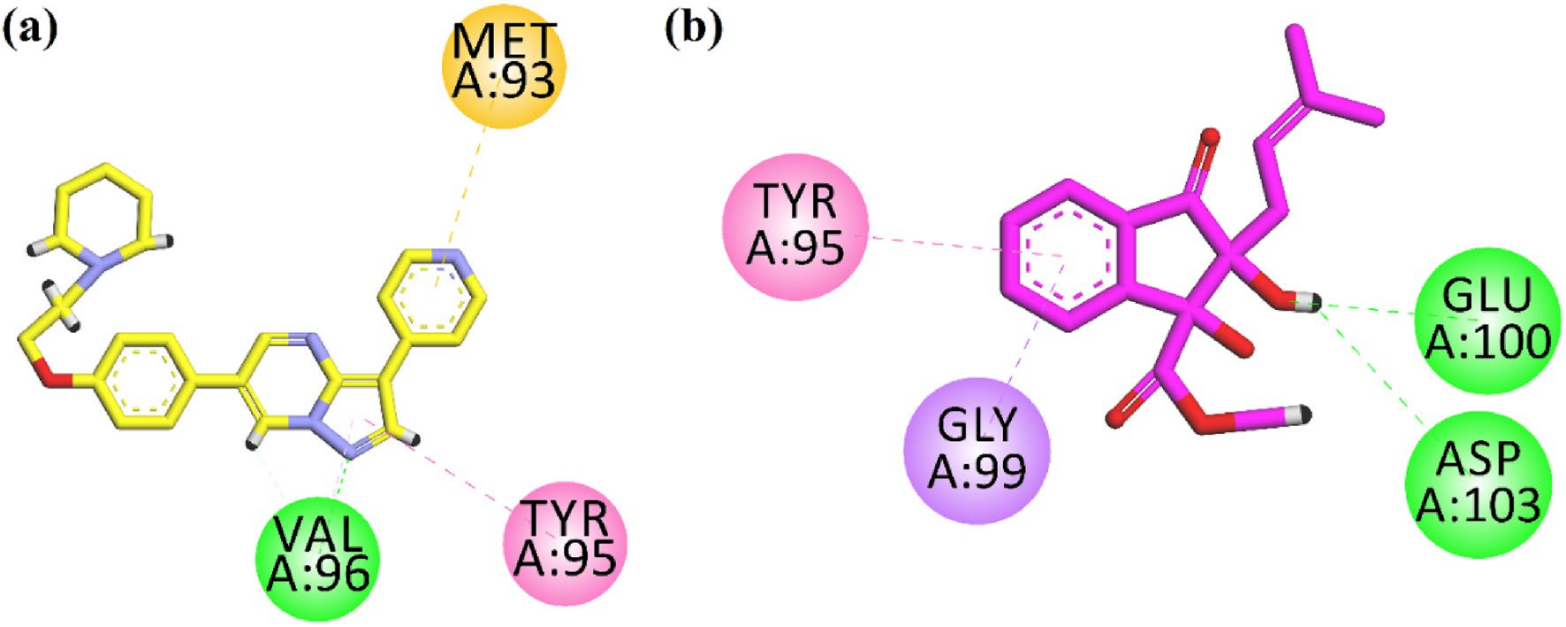
2-D interaction plots of (**a**) native ligand Dorsomorphin and (**b**) isolated indanone in the binding site of AMPK (PDB ID = 3AQV).

Next, we docked the compound into the binding site of the anti-diabetic target AMPK. Figure 5a shows the interaction plot of the native ligand. It interacts with Val96 through hydrogen bond interaction. Tyr95 and Met 93 interact via hydrophobic interactions (Fig. 5a). Isolated indanone derivative forms two hydrogen bond interactions with Glu100 and Asp103. While the phenyl ring forms π–σ interactions with Gly99 and π–π stack interactions with Tyr95 (Fig. 5b). The computed binding energy values of native ligand; 6-[4-(2-piperidin-1-ylethoxy)phenyl]-3-pyridin-4-ylpyrazolo[1,5-a]pyrimidine, also known as Dorsomorphin. It is a potent, selective, reversible, and ATP-competitive inhibitor of AMPK, and indanone derivative in the binding site of AMPK are − 8.39 and − 7.06 kcal/mol, respectively.

### Molecular dynamics simulation

The docking study is static, single-picture explanation of the target protein–ligand complex interaction. For further detailed analysis of protein–ligand complex (ICX2 and 1EQG) molecular dynamics simulations was performed using Desmond software^1^. The best pose of the docking study based on interaction and S-score was selected to investigate the structure and dynamics of target protein and ligand. The conformational changes, flexibility, dynamics of target proteins (1CX2 and 1EQG), and, most importantly, the effect of ligand binding to the proteins were evaluated in terms of root mean square deviation (RMSD) and root mean square fluctuation (RMSF). Ligand RMSD, radius of gyration (rGyr): intramolecular hydrogen bond (intraHB), Molecular surface area (MolSA), Solvent accessible surface area (SASA), Polar surface area (PSA) parameter were used to check and illustrate the consistency and orientation of our compound deep inside the receptor site of target protein (1CX2 and 1EQG).

Additionally, RMSF graph and analyses were used to demonstrate the stability of the structure, atomic dynamics, and amino acid versatility during the interaction of target protein (1CX2 and 1EQG) with a hit.

**Figure 6:**
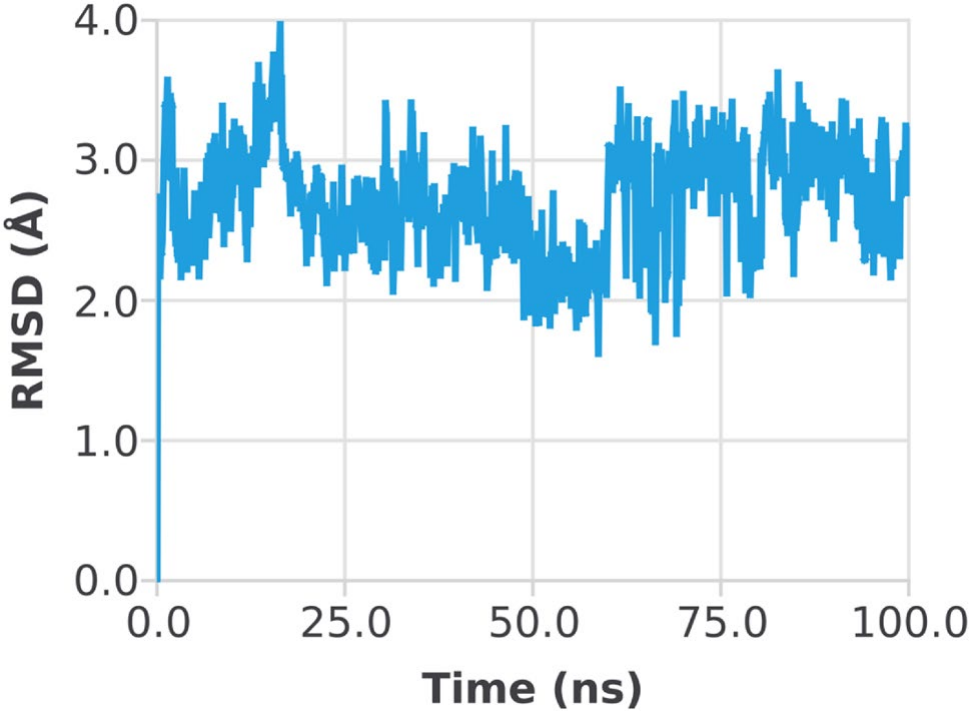
Root mean square deviation (RMSD) plot of 1CX2 protein–ligand (complex).

The RMSD of the 1CX2–ligand complex revealed a little deviation at about 60 ns, where the deviation was between 2 and 3 Å; after that, the system became equilibrated throughout the experiment (Fig. 6). When the trajectories were examined, it was discovered that both of the structures 1CX2 and 1EQG had a stable state, ligands remained deep inside the binding cavity (receptor site) and produced substantial interactions, and that the backbones were consistent. Conformational adjustments could be the reason for the variance in results.

**Figure 7:**
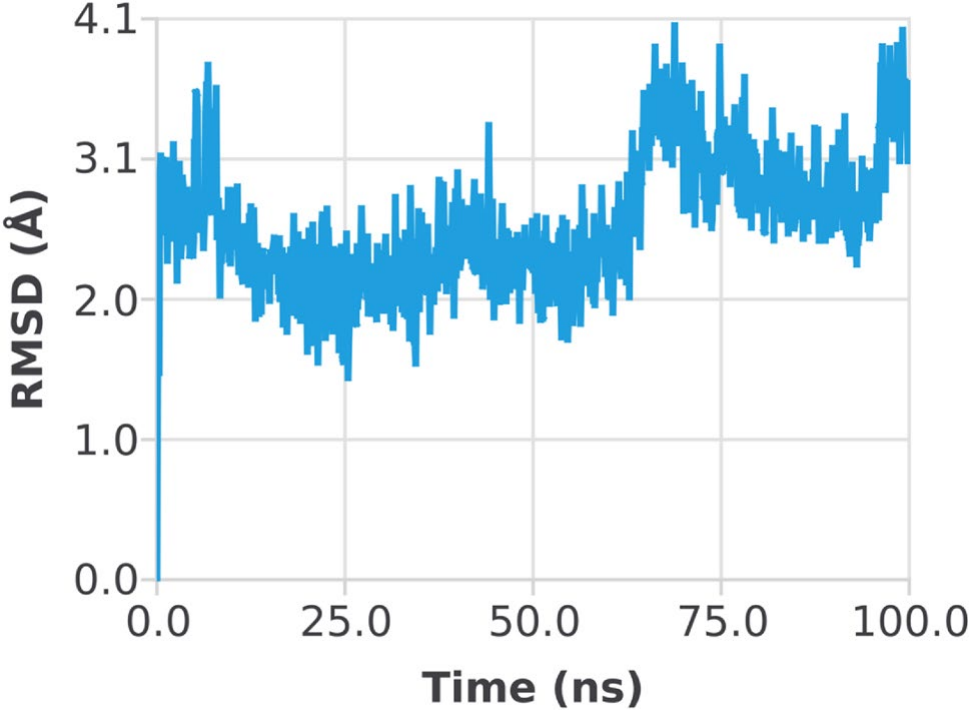
Root mean square deviation (RMSD) plot of 1EGQ–ligand (complex).

**Figure 8:**
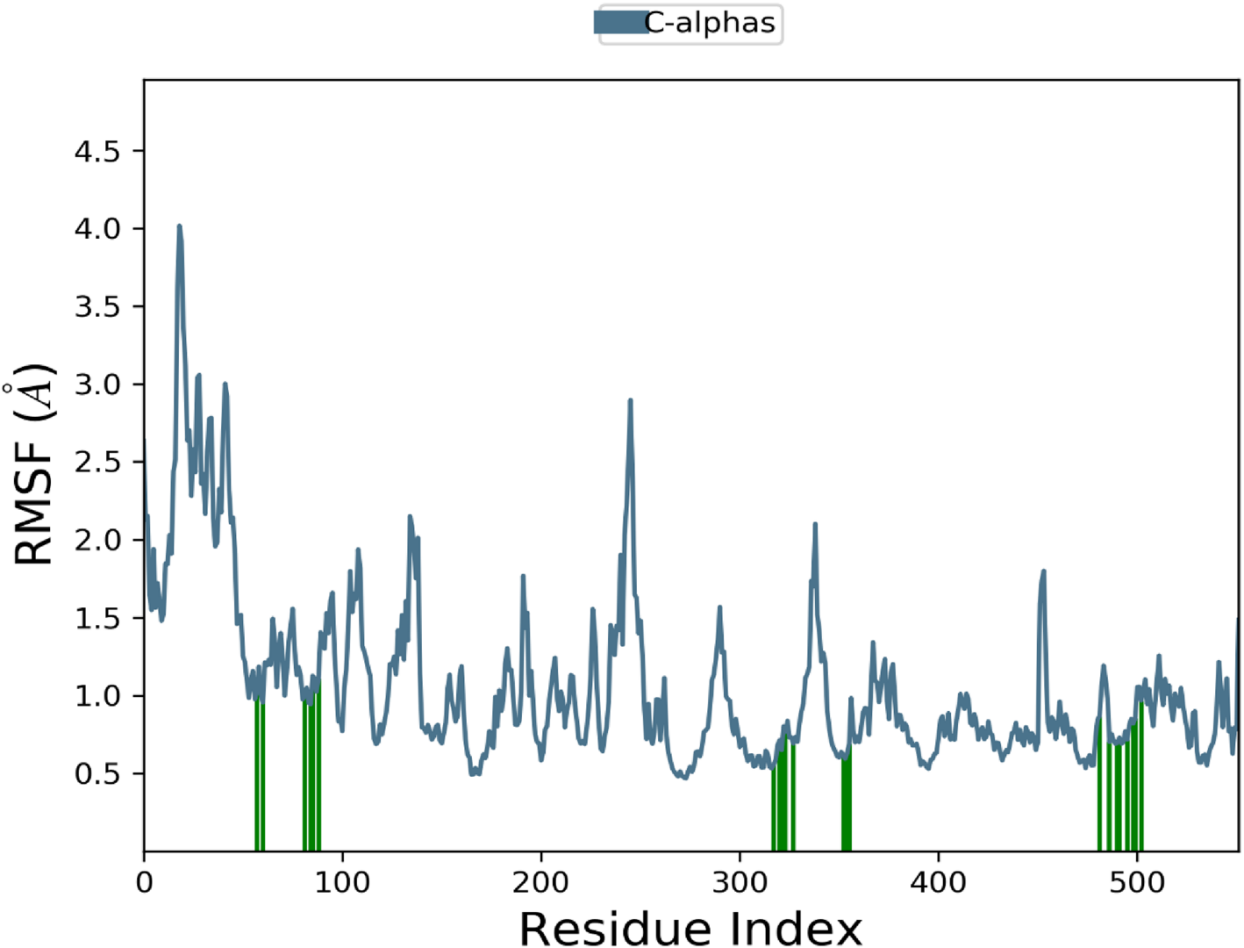
Root mean square fluctuation (RMSF) plot of 1CX2–ligand (complex).

**Figure 9:**
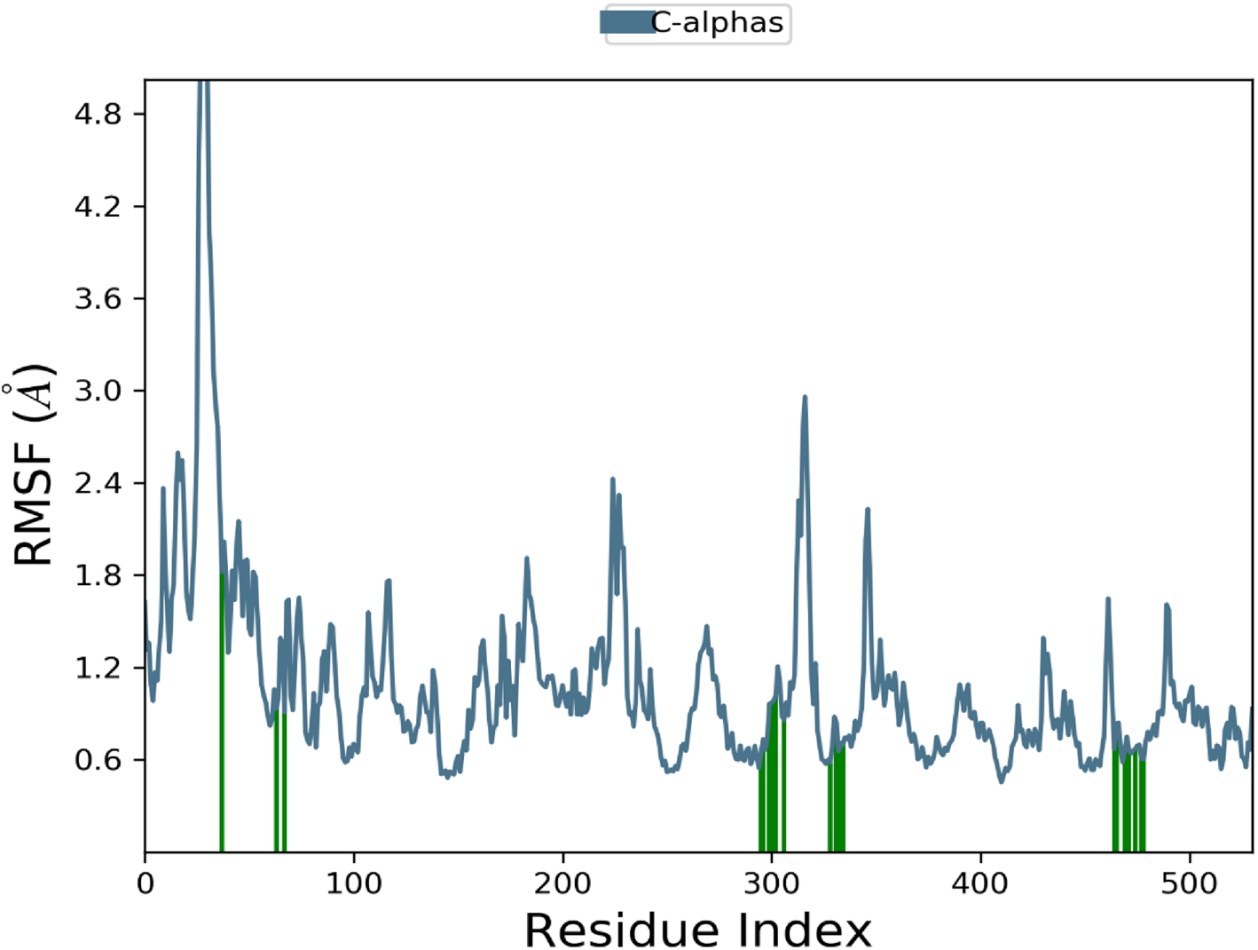
Root mean square fluctuation (RMSF) plot of 1EQG–ligand complex.

The RMSD of the 1EGQ-Complex exhibited a variation from 2 to 3 Å at roughly 60 ns, with a tiny flip from 3 to 4 nm, although overall deviations were within range (Fig. 7). The RMSF graph peaks of 1CX2 shows stability in term of fluctuation in most of the of part of the protein during the simulation but little fluctuation was shown from residues 30 to 50 and 240 to 260. On the other hand, the 1EQG RMSF plot shows slight fluctuation between residues 30–40 and 300–340. The binding of compound for 1CX2 and 1EQG lowered the value of RMSF (< 3 Å), suggesting that the ligand-enzyme complexes are stable (Figs. 8, 9).

**Figure 10:**
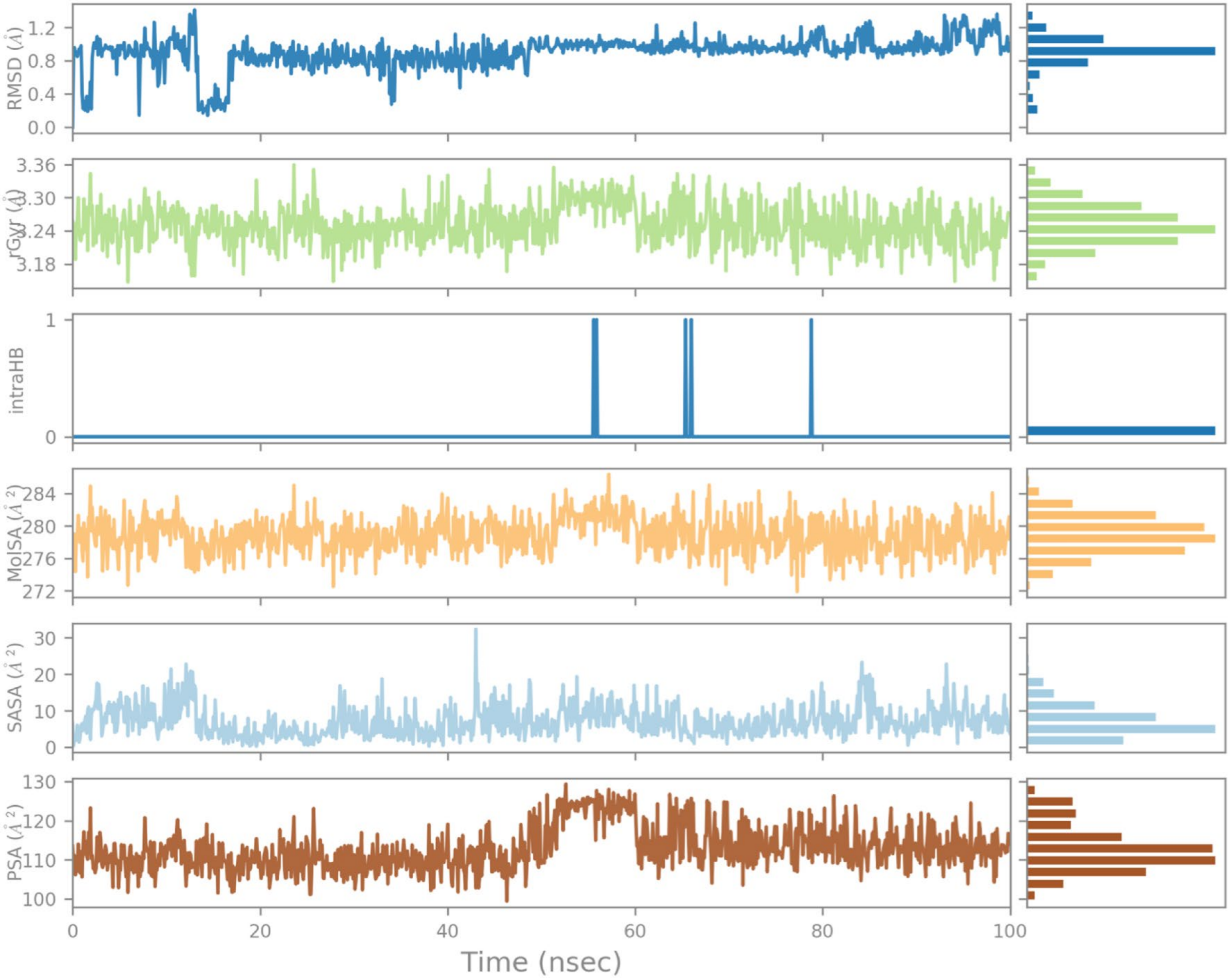
Variation in the ligand’s properties in 1CX2-complex concerning time during the course of 100 ns simulation.

The influence of indanone derivative in target protein was studied in complex with 1CX2. To analyze the position, stability, and orientation of our compound deep inside the receptor site of the target protein, six different parameters were determined. RMSD of ligand was compared with the reference conformation (typically the first frame is used as the reference, and it is regarded as time t = 0). The graph shows stability with some variation from 0–20 ns. The intraHB, MolSA, SASA, and PSA plots also showed the consistency of the ligand during the whole simulation process. The MolSA, SASA, and PSA plots for the ligand were consistent, and curves were smooth throughout the 100 ns simulation. (Fig. 10).

**Figure 11:**
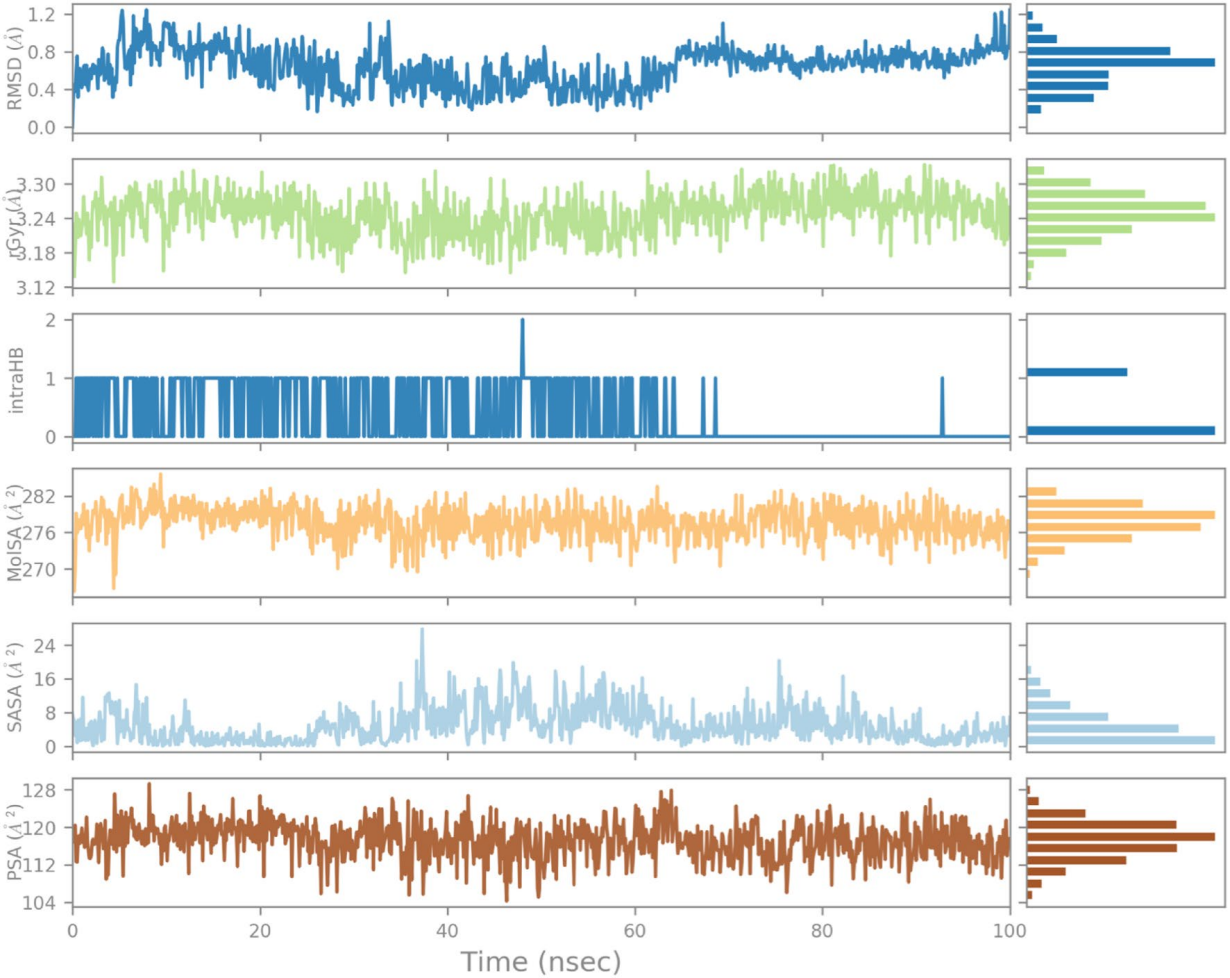
Variation in the ligand’s properties in 1EGQ-complex concerning time during the course of 100 ns simulation.

As shown in Fig. 11, the RMSD of the ligand (indanone derivative) was nearly 1.2 Å from almost 0–20 Å and was consistent till 100 ns. The rGyr value of ligand in the binding was from 3.18–3.30 Å till the end of the simulation. The constant values indicate steady behavior. The intraHB, MolSA, SASA, and PSA plots also showed the consistency of the ligand during the whole simulation process. The MolSA, SASA, and PSA plots for the ligand were consistent, and curves were smooth throughout the 100 ns simulation. Similarly, the RMSD of the ligand in the 1EGQ-complex was roughly 1.2 until 100 ns. Until the completion of the simulation, the rGyr value of the ligand in the binding ranged from 3.18 to 3.30. Stable values suggest consistent behaviour. The intraHB, MolSA, SASA, and PSA graphs further demonstrated the ligand's constancy throughout the simulation phase. The ligand's MolSA, SASA, and PSA graphs did not show a lot of variation, and they remained steady throughout the simulation.

These curves indicated the consistency of the ligands in the binding pocket over the simulation trajectory (Fig. 11).

### In-silico pharmacokinetic predictions

In-silico pharmacokinetic properties were predicted by using the Admet-SAR online server. The results are tabulated in Table 2. The results showed that the indanone derivative is bioavailable and absorbed in the human intestine. Moreover, it is non-carcinogenic and also not able to cause mutagenesis. The acute oral toxicity of the compound is predicted to be in Category III, which is considered safe for human consumption according to the Organization for Economic Cooperation and Development (OECD) guidelines.

**Table 2:** In-silico pharmacokinetic prediction results.

ADMET-predicted profile	Value	Probability
Human intestinal absorption	Positive	0.9913
Human oral bioavailability	Positive	0.6857
Carcinogenicity	Negative	0.9311
Ames mutagenesis	Negative	0.6000
Acute oral toxicity	III	0.4345

## Discussion

Most plant species belonging to the Bignoniaceae genus have been recognized as abundant sources of phytochemicals, with lapachol being widely acknowledged as the most prevalent among them^16,17,19,20^. Extensive scientific investigations have been conducted on lapachol and its derivatives, unveiling their potential anti-inflammatory, anti-malarial, anti-carcinogenic, anti-abscess, anti-ulcer, anti-endemic, termiticidal, and viricidal activities^17,20^. Furthermore, α-lapachol, lapachol, indanone derivative, and peshawaraquinone present in the genus Bignoniaceae have been demonstrated to inhibit the activity of phosphodiesterase 1 and inflammation^21,22^.

The presence of phytochemicals in medicinal plant extract might enhance glucose uptake. Therefore, in this study, the indanone derivative isolated from the root heartwood of *Fernandoa adenophylla* was evaluated for potent anti-inflammatory and anti-diabetic properties. An increase in glucose uptake by yeast cells due to the isolated indanone derivative was noticed in this study. In skeletal muscles, glucose uptake happens due to the accumulation of glucose-transporting molecules through the cell membrane. Leptocytes play a regulatory role in the transportation of glucose by adjusting glucose-transporting molecules in response to elevated insulin levels in the bloodstream^22^. The glucose uptake cell assay is a crucial test utilized for evaluating the in-vitro anti-diabetic properties. The isolated compound effectively boosts and stimulates glucose uptake in peripheral cells^23^. Like standard anti-diabetic medications, isolated compounds also facilitate glucose uptake by cells. Furthermore, these isolated compounds may exhibit anti-diabetic effects through specific mechanisms. In addition, facilitated diffusion serves as a means for glucose to transport across the membrane of yeast cells. As a result, isolated compounds have the potential to enhance glucose uptake through improved metabolism and facilitated diffusion. However, it is crucial to note that further in vivo studies are necessary to verify the binding of the isolated compound to glucose and its subsequent transport.

Scientists often employ various assays, such as RBC membrane stabilization and heat-induced hemolysis of RBC, to assess the anti-inflammatory properties of isolated natural compounds. In our study, a heat-induced hemolysis assay was specifically conducted to evaluate the potential anti-inflammatory properties of isolated indanone derivatives. As detailed in the results section, the indanone derivative exhibited a significant reduction in heat-induced RBC membrane lysis. This inhibitory effect was noted to be in a dose-dependent manner. The highest inhibition of 72.82% was noticed at a concentration of 100 µM. On the other hand, a standard drug (diclofenac sodium) used to inhibit inflammation commercially showed inhibition of 85.71% at the same concentration. The outcomes of this study demonstrate that the indanone derivative significantly stabilized the RBC membrane. This may be due to the binding of the compound with lysosomal enzymes^24^. Inflammation leads to tissue damage, and both chronic and acute inflammations contribute to membrane destabilization^25^. Anti-inflammatory drugs can help prevent membrane destabilization. The shift in consumer preferences towards natural products has prompted scientists to explore safe and cost-effective alternatives to synthetic medications. Over the past decade, isolated natural plant components from their source matrices have proven effective in treating various ailments^26–29^. Heat-induced RBC hemolysis assay provides initial information on lysosomal membrane stabilization, which is thought to be a key regulator during reactions associated with inflammation^27,30^. Anti-inflammatory agents inhibit the release of hemoglobin from the ruptured cell by maintaining the membrane’s permeability, which increases due to high-temperature application^31^. In a nutshell, the outcomes of the present study showed that isolated indanone derivatives possessed in-vitro anti-inflammatory and anti-diabetic characteristics in a dose-dependent manner.

Four target enzymes underwent docking studies, where the indanone derivative was placed within the binding site of four molecular targets associated with anti-diabetic and anti-inflammatory properties. The isolated indanone core interacts with all the studied molecular targets via hydrophilic and hydrophobic interactions. In the binding sites of cyclooxygenase isoforms (COX-1 and COX-2), key enzymes converting arachidonic acid to prostaglandins and other lipid mediators interact with the specific amino acid residue of both isoforms, showing that it may be a non-selective inhibitor. However, the computed binding energy values showed that the indanone derivative showed more selectivity towards the COX-2 isozyme, which is beneficial because COX-1 maintains the body’s homeostasis in normal conditions. COX-2, on the other hand, is activated during an inflammatory reaction when an immune response develops.

Moreover, it showed good interactions and binding affinity value close to the native ligand in the TNF-α binding site, a key target for developing drugs for many inflammatory-related diseases such as rheumatoid arthritis. The binding energy value also confirmed the dual anti-inflammatory and anti-diabetic effects in the binding site of the anti-diabetic target AMPK.

## Materials and methods

### Chemicals

In this study, all the chemicals, standards, and reagents were purchased from Sigma and Merck and were of analytical grade. Specifically, Diclofenac sodium, Metformin, Baker’s yeast, PBS-solution, and EDTA were purchased from Sigma. However, dichloromethane, methanol, chloroform, ethyl acetate, n-Hexane, butanol, and silica gel column were procured from Merck.

### Plant material collection

The root heartwood of *Fernandoa adenophylla* was collected from different locations at the University of Peshawar, KPK, Pakistan. Dr. M. Ilyas from the University of Sawabi (UOS) KPK identified the specimens, and the voucher specimen (UOS/Bot761) was placed in the Herbarium of UOS, KPK, Pakistan. The Experimentation Ethical Committee of the Department of Chemistry at the University of Swabi on using plant samples approved the experimental protocol. Moreover, it was carried out in strict compliance with the National Research Council guidelines on the care and use of experimental plants.

### Extraction, fractionation, and isolation

**Figure 12:**
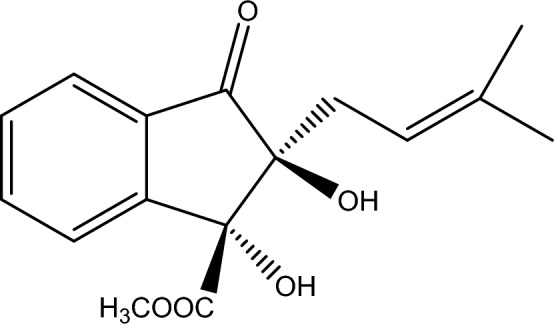
Chemical structure of methyl (1R trans, 2S trans)-1,2-dihydroxy-2-(3-methylbut-2-en-1-yl)-3-oxo-2,3-dihydro-1H-indene-1-carboxylate isolated from *Fernandoa adenophylla.*

Roots heartwood (1 kg) was dried under shade and subjected to a commercial grinder for size reduction. Further, this powdered plant sample was extracted using 20 L methanol for 14 days. The mixture was then filtered and vacuum-dried to gain 80 g of crude extract. The obtained methanolic crude extract was later fractioned using n-hexane, dichloromethane, ethyl acetate, and methanol. Afterward, successful concentration was achieved through a rotary evaporator, and resultant fractions were ethyl acetate (6.53 g), dichloromethane (5.23 g), n-hexane (2.74 g), and methanol (10.91 g). For isolation of indanone derivative, the methanolic fraction (10.91 g) was assessed through column [silica gel column-60 (0.0062–0.200 mm; Merck)] chromatographic analysis. The methanolic fraction was eluted through varied proportions of chloroform and methanol, which resulted in 114 sub-fractions. Depending upon comparative thin layer chromatographic profiling, sub-fraction two was again eluted by chloroform: methanol (7:3) through column chromatography, obtaining the indanone derivative. The chemical structure of the indanone derivative was identified by comparison of previously reported compounds (12); see (Fig. 12).

### In-vitro anti-inflammatory activity

Heat-induced hemolysis assay was performed to determine the in vitro anti-inflammatory characteristics of indanone derivative isolated from root heartwood of *Fernandoa adenophylla*. For this purpose, the effect of varying concentrations (10–100 µM) of isolated compound (indanone derivative) on hemolysis of human RBC was evaluated by following the methods of Shams et al.^24^. Blood (5 mL) was drawn from healthy volunteers who had not been administered anti-inflammatory medications for ten days before this experiment. Blood was transferred in an anti-coagulant (EDTA) containing flacon tube followed by centrifugation (3000 rpm; 5 min). After centrifugation, precipitates were washed using an isosaline solution. This process of centrifugation and washing was repeated till the appearance of clear supernatant. Suspensions (10%) were prepared in an isotonic solution from pellets having RBC. Following reaction procedures were used for the preparation of the control reaction mixture [100 µL and 900 µL of blood suspension (10%) and PBS solution (distilled water: 20 µL, PBS; 880 µL), respectively], standard reaction mixture [100 µL and 900 µL of blood suspension (10%) and various concentrations of diclofenac sodium (DS) + PBS solution (DS: 10 µL + PBS; 890 µL, DS: 20 µL + PBS; 880 µL, DS: 30 µL + PBS; 890 µL, and DS: 80 µL + PBS; 820 µL, etc.), respectively] and test samples reaction mixture [100 µL and 900 µL of blood suspension (10%) and various concentrations of isolated compound (indanone derivative) + PBS solution (indanone derivative: 10 µM + PBS; 890 µL, indanone derivative: 20 µM + PBS; 880 µL, indanone derivative: 30 µM + PBS; 890 µL, and indanone derivative: 80 µM + PBS; 820 µL, etc.), respectively]. The mixtures were incubated at 54 °C for 30 min, then centrifuged at 5400 rpm for 5 min. All samples were taken in triplicate, and a Spectrophotometer analyzed absorbency at 560 nm.

The formula used for the determination of the percentage of inhibition (H) of RBC lysis:

$$\%\,\, inhibition (H) = \left[\frac{Abs\,\, control -Abs\,\, sample}{Abs\,\, control}\right] \times 100$$

### In-vitro anti-diabetic activity

For evaluating the in-vitro anti-diabetic activity of varied content (5–100 µM) of isolated indanone derivative and reference standard (Metformin)^32–34^, glucose uptake by baker’s yeast assay was performed according to the procedure mentioned by Shams et al.^25^ with slight modification. It was centrifuged after thoroughly washing the baker’s yeast (4200 rpm/5 min). Later, the baker’s yeast suspension (10% v/v) was prepared by using distilled water. Different experimented contents (5–100 µM) of indanone derivative and reference standard were incubated at 37 °C for 10 min after mixing with 1 mL of glucose solution (5 mmol/L). After centrifugation, 0.1 mL of yeast solution was mixed and incubated for 60 min. In the end, the sample tubes were placed in a centrifugation machine for centrifugation (5 min; 3000 rpm), and the supernatant was separated and used to calculate glucose content. The experiment was performed in triplicate. The percentage increase in glucose uptake in the cells of yeast was measured using the given formula:

$$\%\,\, glucose\,\, uptake = \left[\frac{Abs\,\, control- Abs \,\,sample}{Abs\,\, control}\right]\times 100$$

### Alpha (α) Amylase Inhibition Assay

α-Amylase is an enzyme that breaks polysaccharides alpha bonds to give glucose and maltose. Because it raises blood glucose levels, inhibiting it lowers the glucose level in the body.

To reveal the inhibition of α amylase by standard drugs as well as by compounds, the method of Shai et al. was followed. Different compounds and typical drug concentrations were made, and 2 U/mL porcine pancreatic amylases (500 µL) in phosphate buffer were incubated for 20 min at 37 °C. To phosphate buffer (100 mM), 1% starch of 250 µL volume was added. After that, it was added to the reaction and then incubated for 1 h at 37 °C. Following that, for 10 min, 1 mL of di-nitro-salicylate reagent was added, and the mixture was boiled. At 540 nm, the absorbance was calculated and recorded. Control was used to find out the inhibitory impact. The following formula was used to measure the percent inhibition.

$$\%\,\, inhibition=\left[\frac{Abs\,\, control-Abs\,\, sample}{Abs\,\, control}\right]\times 100$$

### Docking studies

To evaluate the inhibition potential of our compound as anti-inflammatory and antidiabetic. The synthesized compound was docked into the active site of target proteins including TNF- α, COX-2, AMPK and COX-1. The crystal structures of our target proteins were retrieved from protein data bank (PDB) using four latter access code (PDB id). The pdb ids for TNF- α, COX-2, AMPK and COX-1 is 2AZ5, 1CX2, 3AQV and 1EQG, respectively. The synthesized compound and target proteins were prepared (protonated and minimized) according to our previously standard reported method^35,36^. After the downloaded enzymes were ready, to validate the authenticity of our docking method/procedure the standard/native ligand of target protein was redocked in active site of respective enzymes. Docking protocols with RMSD values of less than 2.0 Å were used for further studies. Docking rums were carried out by using default parameters. The Molecular Operating Environment (MOE) software preparation module were used for the three-dimensional protonation and preparation of target proteins. The Amber 10EHT force field was used to minimize the energy of our target proteins and compound with 0.1 and 0.00001 gradient, respectively. For docking simulations, active sites were determined with 10 Å of the native ligands. Docking parameters were validated by using the redocking of native ligands into the binding sites of their respective enzymes. Another key parameter of our docking method triangle matcher was used with London dG/Affinity dG as scoring function while GBVI/WSA function used as rescoring-2. For the protein–ligand interaction and docking study profile top ten conformations were allowed to produce for every ligand. To analyzed the docking results and interaction of ligand deep inside the receptor site of target proteins the discovery studio visualizer (DSV) software were used.

### Molecular dynamic (MD) simulation

MD simulation is a sophisticated computer approach for studying the behavior and characteristics of molecules and materials at the atomic level. Schrödinger's Desmond is a software programme for MD simulations of biological systems such as proteins, nucleic acids, and membranes.

The stability of protein–ligand interactions was explored using molecular dynamics (MD) simulations. MD simulations were specifically performed on the most stable complexes with high binding energies. Desmond MD simulations were used to investigate the dynamic binding behavior and binding stability of docked protein–ligand complexes. With NPT ensembles, the simulation was conducted for 100 ns at 1 atm and 300k. A more extensive discussion of the process is available elsewhere^37–40^.

### In-silico pharmacokinetic predictions

In-silico pharmacokinetic properties were predicted by using the Admet-SAR online server. SMILES strings were submitted to the online server, and the properties were predicted^41^.

### Statistical analysis

Results were confirmed as mean ± SD and compared with the control group using SPSS 22.0 software. Data were subjected to ANOVA followed by Tukey’s multiple comparison tests to determine significant differences using SPSS 22.0 software. P < 0.05 was considered statistically significant.

## Conclusions

The present finding reports of the isolation of indanone derivative from the Roots heartwood of *F. adenophylla.* The isolated compound, namely methyl (1R trans, 2S trans)-1,2-dihydroxy-2-(3-methylbut-2-en-1-yl)-3-oxo-2,3-dihydro-1H-indene-1-carboxylate showed significant in-vitro anti-diabetic and anti-inflammatory effects. Our findings validate the traditional use of *Fernandoa adenophylla* in treating various ailments. Accordingly, the idanone derivative showed significant anti-inflammatory and anti-diabetic properties. It thus may be a potent candidate for drug discovery subject to further detailed in-vivo and clinical studies. Docking studies on various molecular targets of the disease showed that the isolated compound might act by interacting with the critical amino acid residues of 5′ AMPK, COX-1, and COX-2. Moreover, it showed good interactions in the TNF-α binding site, a key target for developing drugs for many inflammatory diseases such as rheumatoid arthritis. Thus, our finding validated the traditional usage of *Fernandoa adenophylla,* and the tested molecule might be further screened as a candidate for clinical studies. The molecular dynamics simulation results (RMSD and RMSF) of complexes of target proteins (COX-1 and COX-2) and selected compounds are stable and in an acceptable range with slight variation from the average. Overall, MDS results show that candidate compounds have good inhibition against COX-2 compared to COX-1.

## Data Availability

All data generated or analysed during this study are included in this published article.
